# Laboratory Diagnoses

**Published:** 1907-01-05

**Authors:** 


### The Hospital

A Journal of

The Medical Sciences and Hospital Administration.

Vol. XLI.—No. 1,060. SATURDAY, JANUARY 5, 1907.

### LABORATORY DIAGNOSES.

Perhaps the most impressive feature in the recent history of practical medicine has been the application of laboratory investigations to the art of diagnosis. It is no longer sufficient to hear from the patient an account of his symptoms and to determine his bodily condition by various methods of physical examination. A more searching and penetrating scrutiny is often demanded, and this is usually accomplished by investigations conducted in the laboratory. New discoveries in physics, in chemistry, and in biology, have been applied in the domain of practical medicine, and new methods of diagnosis have thus come into operation. The examination of the various excretions and of the blood, and the application of the *x*-rays, may be mentioned as examples of recent specialised processes which are now constantly called upon to aid the work of the physician and surgeon. As a result of these and similar developments there have come into existence a number of observers who conduct their diagnostic work apart, largely, from the person and symptoms of the individual patient. Equipped with technical knowledge and experience, these clinical pathologists, as they are often called, are frequently able to submit statements of fact, of great value and significance, to the medical practitioner in charge of the patient. The clinical study of the sick man is in this way enlarged, and the art of medicine is made more exact and more confident.

No one with any actual experience of the difficulties of diagnosis presented by many cases, will, for a moment, question the enormous advantages which have accrued from the more careful analytical processes that are now at the disposal of the practitioner. To state these in detail would be to write a large part of recent medical history. Yet, as in most human affairs, the gain is not altogether an unqualified one, and with it there have come new dangers and new risks. Among these may be mentioned a tendency to leave the last word, even in the diagnosis of an individual case, to the laboratory worker. His results appear so clean-cut and so confident, that in them, not only the truth, but the whole truth, would seem to be contained. The true position, of course, is that these results should be added to the other facts of the case, and that only after a consideration of the whole should the diagnosis be framed. The patient remains, and ever must remain, the central feature of the problem, and whilst all possible aids must be invoked, it is at the bedside and not in the laboratory that the decision of the issue has to be taken. Thus, to whatever degree laboratory methods and processes may be cultivated, there must be no weakening of the demand for the cultivation of careful and minute clinical observation and knowledge.

Another aspect of laboratory practice well worthy of consideration is the bearing which such practice has upon the responsibility of the individual practitioner. It must ever be remembered that the relationship of the patient to his medical adviser is a confidential and personal one. The practitioner in assuming that relationship tacitly accepts this position, and the responsibility which it involves. Is he, in such circumstances, fully justified in expressing an opinion and in advising the patient, when he acts, not upon first-hand information, but upon information conveyed to him by another practitioner who has no personal knowledge of the patient and only a very limited view of the facts to be discussed? Especially acute does such a question become when the report on some particular test rests, not on the authority of some recognised expert worker, but merely on the statement of an unknown laboratory official. In such circumstances the tests have been applied and the results obtained by some person of whose professional value the practitioner in attendance has no means of judging; and this, in view of the personal responsibility of the practitioner to his patient, would appear to raise an ethical question of some delicacy. Even at the best the laboratory worker is not under the same conscious pressure of responsibility as is the clinical adviser, and amidst large numbers of specimens there are dangers of confusion and mishap which can hardly be avoided. It is therefore fair to repeat that the new methods have brought with them dangers and risks as well as advantages and aids.

It remains to be asked whether anything can be done to avoid the accidents possible to the laboratory, while at the same time retaining its undoubted value. That laboratory methods and processes are now an essential part of the medical art must be recognised, and the recognition need not be a grudging one. But it seems a necessary conclusion that the individual practitioner must be competent, not only to appreciate, but to check, the results of such processes. Unfortunately the tendency just now appears to be to leave to the laboratory even many tests which formerly were applied in the surgery or the consulting-room. But if personal authority and responsibility are to be maintained, the movement of affairs ought to be in exactly the opposite direction. Allowing that there are a few modern methods which demand much time and a highly technical equipment, there are still many which can be very easily accomplished. And thus, so far from leaving more and more to the laboratory, it is, in our view, the duty of the practitioner steadily to enlarge his own personal capacity in these modern directions. Even in instances where this is not possible, it seems to us essential that the immediately responsible practitioner shall see personally the results of the tests, and shall not take important decisions on hearsay evidence and on reports which are not authenticated by some recognised and responsible medical authority. Laboratory methods have great value, but they ought to be used, not to contract, but to enlarge, the area of personal observation of the practitioner.

